# Targeting Heat Shock Proteins Mitigates Ventilator Induced Diaphragm Muscle Dysfunction in an Age-Dependent Manner

**DOI:** 10.3389/fphys.2016.00417

**Published:** 2016-09-27

**Authors:** Hannah Ogilvie, Nicola Cacciani, Hazem Akkad, Lars Larsson

**Affiliations:** ^1^Basic and Clinical Muscle Biology, Department of Physiology and Pharmacology, Karolinska InstitutetStockholm, Sweden; ^2^Department of Clinical Neuroscience, Clinical Neurophysiology, Karolinska InstitutetStockholm, Sweden

**Keywords:** diaphragm, ventilator-induced diaphragm dysfunction, aging, BGP-15, heat shock protein

## Abstract

Intensive care unit (ICU) patients are often overtly subjected to mechanical ventilation and immobilization, which leads to impaired limb and respiratory muscle function. The latter, termed ventilator-induced diaphragm dysfunction (VIDD) has recently been related to compromised heat shock protein (Hsp) activation. The administration of a pharmacological drug BGP-15 acting as a Hsp chaperone co-inducer has been found to partially alleviate VIDD in young rats. Considering that the mean age in the ICU is increasing, we aimed to explore whether the beneficial functional effects are also present in old rats. For that, we exposed young (7–8 months) and old (28–32 months) rats to 5-day controlled mechanical ventilation and immobilization with or without systemic BGP-15 administration. We then dissected diaphragm muscles, membrane–permeabilized bundles and evaluated the contractile function at single fiber level. Results confirmed that administration of BGP-15 restored the force-generating capacity of isolated muscle cells from young rats in conjunction with an increased expression of Hsp72. On the other hand, our results highlighted that old rats did not positively respond to the BGP-15 treatment. Therefore, it is of crucial importance to comprehend in more depth the effect of VIDD on diaphragm function and ascertain any further age-related differences.

## Introduction

Mechanical ventilation is a life-saving intervention in critically ill intensive care unit (ICU) patients with respiratory failure. However, there is increasing evidence that assisted or controlled mechanical ventilation (CMV) has significant negative effects on diaphragm muscle function that elicits the condition known as ventilator-induced diaphragm dysfunction (VIDD) (Vassilakopoulos and Petrof, 2004). Since VIDD has a strong impact on the ability of patients to be successfully weaned from the ventilator, it is therefore of critical importance to understand the effect of mechanical ventilation on diaphragm function (Laghi et al., 2003; Haitsma, 2011). A substantial reduction in diaphragm muscle force-generation capacity has been reported in previous studies using young mechanically ventilated baboons, rodents and piglets (Powers et al., 1985; Le Bourdelles et al., 1994; Anzueto et al., 1997; Radell et al., 2002; Ochala et al., 2011; Corpeno et al., 2014). Although the supportive therapy in ICUs has greatly improved during the last decades, recent epidemiological studies have shown that older ICU patients are at greater risk of developing lung diseases and VIDD (Rubenfeld et al., 2005). Detailed information on the effects of aging and mechanical ventilation is limited, even though the age-related muscle wasting, termed “sarcopenia” is known to be detrimental for diaphragm force production in small mammals (Gosselin et al., 1994; Criswell et al., 1997) and humans (Polkey et al., 1997).

Heat shock proteins, are highly conserved molecules found in all cellular organisms. Their primary role is to chaperone, transport and fold proteins when cells are exposed to different types of stress (Senf et al., 2008). The protective role of Hsp's against immobilization-induced limb muscle wasting has been reported in young animals (Aare et al., 2004; Banduseela et al., 2009). The Hsp70 family constitutes the most conserved and best-studied class of Hsp's. Hsp70 promotes protein folding and has been suggested to prevent and/or repair stress-induced protein damage. The stress-inducible isoform of Hsp70 (Hsp72) is generally not expressed in unstressed cells; however, upon exposure to stressful conditions, Hsp72 is highly inducible. During conditions of stress, Hsp72 is thought to bind to damaged and miss folded polypeptides and further facilitate repair (Lindquist and Craig, 1988). In addition, it has several additional roles, including the regulation of various cell signaling pathways related to cell growth and inflammation (Nollen and Morimoto, 2002). At the skeletal muscle level, its specific up-regulation maintains muscle fiber integrity and facilitates muscle regeneration and recovery (Senf, 2013). The first evidence of Hsp72's involvement in regulating skeletal muscle size was through the use of muscle specific Hsp70 transgenic mice (McArdle et al., 2004). Furthermore, Hsp72 has been seen to negatively regulate the Forkhead BoxO (FoxO) and Nuclear Factor κB (NF-κB) pathways, which are activated during numerous conditions of muscle atrophy (Hunter and Kandarian, 2004; Sandri et al., 2004; Senf et al., 2010).

Aged organisms exhibit a greatly decreased ability to produce/activate Hsp's in response to stresses, which correlate with the increase in morbidity and mortality of aged organisms (Heydari et al., 1994). Hsp72 has been reported to be decreased in both adults and aged rats during periods of muscle disuse (Dodd et al., 2009). Hence, aging and a wide variety of pathophysiological and physiological conditions can be linked to membrane changes which modulate membrane-controlled molecular switches and consequently a dis-regulation of Hsp expressions (Criswell et al., 2003). Based on these evidences, we initially hypothesized that a drug that would directly target/up-regulate Hsp's would therefore be beneficial, with potentially a greater positive effect in old organisms.

The non-toxic hydroxylamine (HA) derivatives amplify the expression of Hsp's induced by mild physical or pathophysiological stresses, offering unique drug candidates, potentially enhancing Hsp expression in diseased cells, without significantly affecting healthy cells (Vigh et al., 2007; Gombos et al., 2011). There is growing evidence that the production of Hsp's is triggered by the exposure to different kinds of environmental stress conditions and is linked to changes in lipid composition and in the architecture of membranes (Vigh et al., 1998). A chaperone co-inducer is a substance that cannot induce Hsp's by itself, but can enhance Hsp induction in combination with other mild stresses (Gombos et al., 2011). It has been shown that the non-proteo-toxic, lipid interacting BGP-15 [O-(3-piperidino-2-hydroxy-1-propyl) nicotinic amidoxime dihydrocholride], is capable of Hsp co-induction in the absence of the production of unfolded proteins (Literati-Nagy et al., 2012). The Hsp70 chaperone co-inducer BGP-15 was recently shown to have a muscle sparing effect and improve muscle architecture, strength and contractile function in severely affected diaphragm muscles in in mdx mice (Gehrig et al., 2012), as well as in young rats exposed to long-term mechanical ventilation (Salah et al., 2016). What happens in old animals remains to be studied and according to our hypothesis, we believe that BGP-15 would have a potential beneficial effect in old mechanically ventilated rats. Hence, the main aim of this pilot study was to investigate the impact of BGP-15 on the diaphragm contractile dysfunction in response to 5-day CMV in young adult (7–8 months) and old (28–32 months) female 344 Brown Norway (F344-BN) hybrid rats.

## Materials and methods

### Animals

The diaphragm samples were collected from 17 F344-BN hybrid rats, 7–8 months (young, *n* = 10) and 28–32 months (old, *n* = 7). The rats were obtained from the National Institute of Aging (NIA Bethesda, MD). The rats were anesthetized, mechanically ventilated and paralyzed by neuromuscular blockade (α-cobra-toxin) for durations varying from 0 days (control) to 5 days. The rats were randomly classified into three subgroups within young and old. 4 young rats and 2 old rats, at time 0 days, 3 young, 2 old rats at time 5 days and 3 young, 3 old rats at time 5 days with BGP-15.

Animals were maintained in protein and fluid balance; (1) Intra-arterial solution: Alfa Cobra toxin (Biotoxins, St. Cloud, Florida, FL, USA), Potassium chloride, Oxacillin (Fresenius Kabi, Uppsala, Sweden), Heparin (Leo, Bellerup, Denmark), Ringers (Potassium Chloride, Calcium Chloride, Sodium Chloride, Lactate, (Fresenius Kabi)), Water; (2) Intra-venous solution: Oxacillin (Fresenius Kabi), Ringers, Water, Glucose (Fresenius Kabi) Vamin, (Fresenius Kabi) and Intralipid, (Fresenius Kabi), and 40 mg/kg/day BGP-15 (N-gene). The sham-operated control animals underwent the same interventions as the experimental group, but they were not pharmacologically paralyzed with α-cobra-toxin. That is, sham operated-controls were anesthetized (isoflurane), spontaneously breathing, given intra-arterial and intra-venous solutions, and sacrificed within 2 h after the initial anesthesia and surgery.

The experimental model has previously been described in detail (Dworkin and Dworkin, 2004) and modified to optimize studies of skeletal muscle. Briefly; (1) Electrocardiogram (ECG) electrodes were implanted subcutaneously. (2) An aortic catheter (28-gauge Teflon) was inserted via the left carotid artery to record arterial blood pressure. (3) A 0.9 mm Renathane catheter was threaded into the left jugular vein to administer parenteral nutrition. (4) Three subcutaneous electroencephalogram (EEG) needle electrodes were placed into the skull above the right and left temporal lobes, and a third reference electrode was placed on the neck. (5) The rat was placed on a heating pad to maintain body temperature; temperature was measured by a vaginal thermistor and servo-regulated at 37°C. (6) A silicone cannula was inserted in the urethra to continuously record urine output. Rats are ventilated through a per os coaxial tracheal cannula at 72 breaths/min with an inspiratory and expiratory ratio of 1:2 and a minute volume of 180–200 ml and gas concentrations of 49.5% O_2_, 47% N_2_, and 3% CO_2_, delivered by a precision (volume drift < 1%/wk) volumetric respirator. Intermittent hyperinflations (6 per h at 15 cmH_2_O), positive end-expiratory pressure (1.5 cmH_2_O), and expiratory CO_2_ monitoring are continuous. Neuromuscular block are induced on the first day (100 μg iv. α-cobra-toxin) and maintained by continuous IV infusion (250 μg/day). Mechanical ventilation starts immediately after the NMB induction. Experiments were terminated after 0 days (control) and 5 days (experimental). During surgery or any possible irritating manipulation, the anesthetic isoflurane level is at >1.5%. In no case did animals show any signs of infections, septicemia, or systemic inflammation. The ethical committee at Uppsala University and Karolinska Institutet approved all aspects of this study.

### Muscle biopsies and permeablisation of fibers

The diaphragm muscle was dissected immediately after euthanasia. One half of the mid-costal diaphragm muscle was frozen in liquid propane cooled by liquid nitrogen and stored at −180°C until further analyses. The other half was placed in relaxing solution at 4°C and bundles of ~50 fibers were dissected free and tied with surgical silk to glass capillary tubes at slightly stretched lengths. The bundles were then treated with skinning solution (relaxing solution containing glycerol; 50:50 v/v) for 24 h at 4°C, after which they were transferred to −20°C. Within 1–2 weeks the muscle bundles were treated with a cryo-protectant (sucrose), removed from the capillaries and snap frozen in liquid nitrogen-chilled propane and stored at −180°C for long term storage.

### Single muscle fiber experimental procedure

On the day of the experiment, bundles were de-sucrosed; transferred to a 2.0 M sucrose solution and subsequently incubated in solutions of decreasing sucrose concentrations (1.5–0.5 M) for 30 min each and finally kept in a skinning solution at −20°C. A single fiber was removed from the muscle bundle and was placed between two connectors, at a length of 1 to 2 mm long. One connector leads to a force transducer (model 400A, Aurora Scientific), and the other to a lever arm system (model 308B, Aurora Scientific). The two extremities of the fiber were tightly attached to the connectors as previously described (Moss, 1979). The apparatus was mounted on the stage of an inverted microscope (model IX70; Olympus). While the fiber segments were in relaxing solution, the sarcomere length was set to 2.65–2.75 μm by adjusting the overall segment length and controlled during the experiment using a high-speed video analysis system (model 901A HVSL, Aurora Scientific). The diameter of the fiber segment between the connectors was measured through the microscope at a magnification of × 320 with an image analysis system prior to the mechanical experiments. Fiber depth was measured by recording the vertical displacement of the microscope nosepiece while focusing on the top and bottom surfaces of the fiber. The focusing control of the microscope was used as a micrometer. Fiber CSA was calculated from the diameter and depth, assuming an elliptical circumference, and was corrected for the 20% swelling that is known to occur during skinning (Moss, 1979). Diameter and depth were measured at three different locations along the length of each fiber and the mean was considered representative of cell dimensions. For the mechanical recording, relaxing and activating solutions contained (in mM) 4 Mg-ATP, 1 free Mg^2+^, 20 imidazole, 7 EGTA, 14.5 creatine phosphate, and KCl to adjust the ionic strength to 180 mM and pH 7.0. The concentrations of free Ca^2+^ were 10^−9^ M (relaxing solution) and 10^−4.5^ M (activating solution), expressed as pCa^2+^ (i.e., -log [Ca^2+^]). Apparent stability constants for Ca^2+^-EGTA were corrected for temperature (15°C) and ionic strength (180 mM). The computer program Fabiato (Fabiato, 1988) was used to calculate the concentration of each metal, ligand, and metal-ligand complex. Immediately preceding each activation the fiber was immersed for 10–20 s in a solution with a reduced Ca^2+^-EGTA buffering capacity. This solution is identical to the relaxing solution except that the EGTA concentration is reduced to 0.5 mM, which results in a more rapid attainment of steady force during subsequent activation. Force was measured by the slack-test procedure (Edman, 1979). This was calculated as the difference between the maximal steady-state isometric force in activating solution and the resting force measured in the same segment while in the relaxing solution. Maximal force production was normalized to CSA (specific force, absolute force (P_0_)/CSA). For contractile measurements a strict acceptance criteria is applied. A muscle fiber was accepted and included in the analyses: (i) if the sarcomere length of a single muscle fiber changed < 0.10 μm between relaxation and maximum activation, (ii) if maximal force changed < 10% from first to seventh activation (Moss, 1979).

### Myosin heavy chain (MyHC) isoform expression

**Figure 1:**
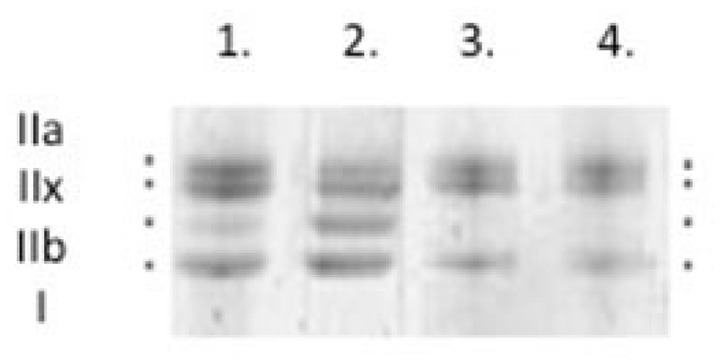
**Typical myosin heavy chain (MyHC) isoform expression from control rat diaphragm muscle was determined by 6% SDS-PAGE**. Types IIa, IIx, IIb, and I.

After mechanical recordings, each skinned fiber or two 10 μm diaphragm cryo-sections from the midcostal region were placed in sample buffer (7.43 ml distilled water, 2.1 ml Glycerol, 1.4 ml 10% SDS, 1.75 of 0.5 M Trisbuffer pH6.8, 0.32 ml bromophenol blue, 32.4 mg Dithiothreitol, 1 ml Leupeptin) in a plastic micro-centrifuge tube and stored at −180°C for subsequent electrophoretic analysis. MyHC isoform composition of fibers was then determined by 6% SDS-PAGE (Figure 1). The acrylamide concentration was 4% (wt/vol) in the stacking gel and 6% in the running gel; the gel matrix included 30% glycerol. Sample loads were kept small (equivalent to ~0.05 mm of fiber segment) to improve the resolution of the myosin heavy chain bands (types I, IIa, IIx, and IIb). Electrophoresis was performed at 120V for 20–22 h with a Tris–glycine electrode buffer (pH8.3) at 10°C (SE 600 vertical slab gel unit, Hoefer Scientific Instruments). The gels were silver-stained and subsequently scanned in a soft laser densitometer (Molecular Dynamics) with a high spatial resolution (50 μm pixel spacing) and 4096 optical density levels.

### Immunoblotting

Ten sections from the midcostal part of the diaphragm were dissolved in SDS buffer (50 mM Tris-HCl, 2%SDS, 0.1% bromophenol blue, 10% glycerol, and 2%β-mercaptoethanol, pH 8.8), heated for 2 min at 95°C and total protein was quantified using the Pierce 660 nm Protein Assembly Assay (Thermo Scientific). Equal amounts of protein were separated on SDS-polyacrylamide gels (Mini-PROTEAN 3 Cell, Bio-Rad Laboratories) at constant 120 volts for 90 min. Acrylamide concentrations were 4 and 12% (w/v) in stacking and running gels respectively, and the gel matrix included 10% glycerol. Separated proteins were transferred to poly(vinylidene difluoride) membranes (Immobilon-P, Millipore). Membranes were blocked with 3% non-fat milk powder in Tris-buffered saline containing Tween 20 for 1 h and incubated overnight at 4°C with Hsp72- (SMC-100B, StressMarq) and actin (sc-1616, Santa Cruz Biotechnology Inc.) primary antibodies and then with HRP secondary antibodies (anti-mouse, GE Healthcare, and anti-goat, Santa Cruz Biotech). Membranes were digitized with ECL 500 Western Blotting Detection Agent Kit (Amersham Biosciences) and imaging system (Odyssey, LI-COR Biosciences). Band densities were quantified with Image Studio Lite analysis software (LI-COR Biosciences) and normalized to actin.

### Total RNA isolation and quantification

Total ribosomal nucleic acid (RNA) was extracted from frozen diaphragm muscle tissue (10–30 mg) using QiagenRNeasy® Mini Kit (Qiagen, Inc., Valencia, CA). Muscle tissue was homogenized using a motor homogenizer (Eurostar Digital, IKAWerke). QIAshredder™ columns (Qiagen Inc., Valencia, CA) were used to disrupt DNA. Total RNA was eluted from RNeasy® Mini columns with 30 μl of RNase-free water. RNA-concentrations were then quantified using nanodrop 1000 spectrophotometer (Thermofisher Scientific, MA, USA).

### Real time polymerase chain reaction (qPCR)

qRT-PCR was used to quantify the mRNA levels for rat atrogin-1, MuRF1. 300 ng of total RNA from diaphragm samples were reverse transcribed to cDNA using Qscript cDNA supermix (Quanta Biosciences, USA). cDNA was amplified in duplicate using MyiQ™ single color real time PCR detection system (Bio-Rad Laboratories, Inc., Hercules, CA, USA). Each reaction was performed in a 20 μl volume with SYBR® Green master mix using the manufactures instructions. The thermal cycling conditions include 95°C for 10 min, followed by 50 cycles of a two-step PCR with denaturation at 95°C for 15 s and a combined annealing and extension step at 60°C for 1 min. Taqman primers were designed using the software Primer Express®(Applied Biosystems, Foster City, CA, USA), ordered from Sigma Aldrich (Sigma-Aldrich St Louis, Missouri, USA). When optimizing each PCR, the PCR products were run on 2% agarose gels to ensure that primer–dimer formation was not occurring. Threshold cycle (Ct) data obtained from running qRT-PCR and analyzed using ΔΔCt, these values were normalized against GAPDH.

### Statistical analysis

Means and standard error of the mean were calculated according to standard procedures. A two-way ANOVA was used for the analysis of the diaphragm single fibers. A one-way ANOVA was used to analyze Hsp72 protein expression, MuRF1 and atrogin-1 gene expression. *p* < 0.05 was considered statistically significant.

## Results

### Effects of 5 days CMV with and without BGP-15 on diaphragm single muscle fiber cross sectional area and diaphragm muscle myosin heavy chain isoform composition

**Figure 2:**
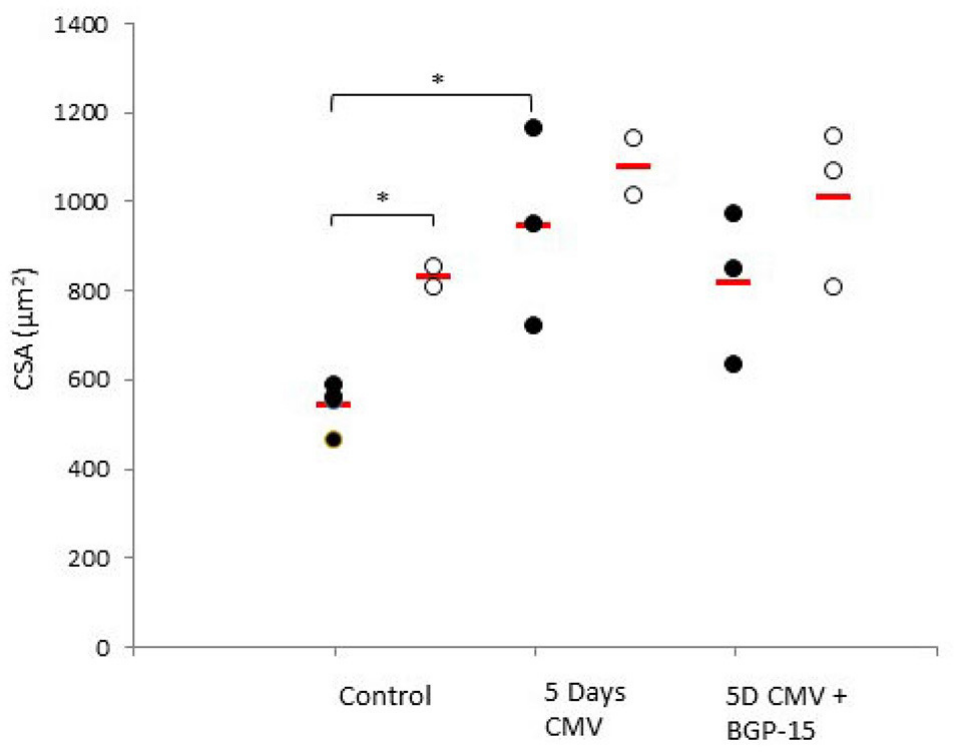
**Individual rat single diaphragm muscle fiber cross sectional area (CSA) measured at fixed sarcomere length in control animals (individual rats: young ***n*** = 4 and old ***n*** = 2) compared with age-matched animals exposed to CMV for 5 days without (individual rats: young ***n*** = 3 and old ***n*** = 2) and with BGP-15 (individual rats: young ***n*** = 3 and old ***n*** = 3)**. Values are averages of individual data from the different animals and the red line indicates the average for each group. (Filled circles, young; open circles, old; red line, group average). Significance level, ^*^p < 0.05.

**Figure 3:**
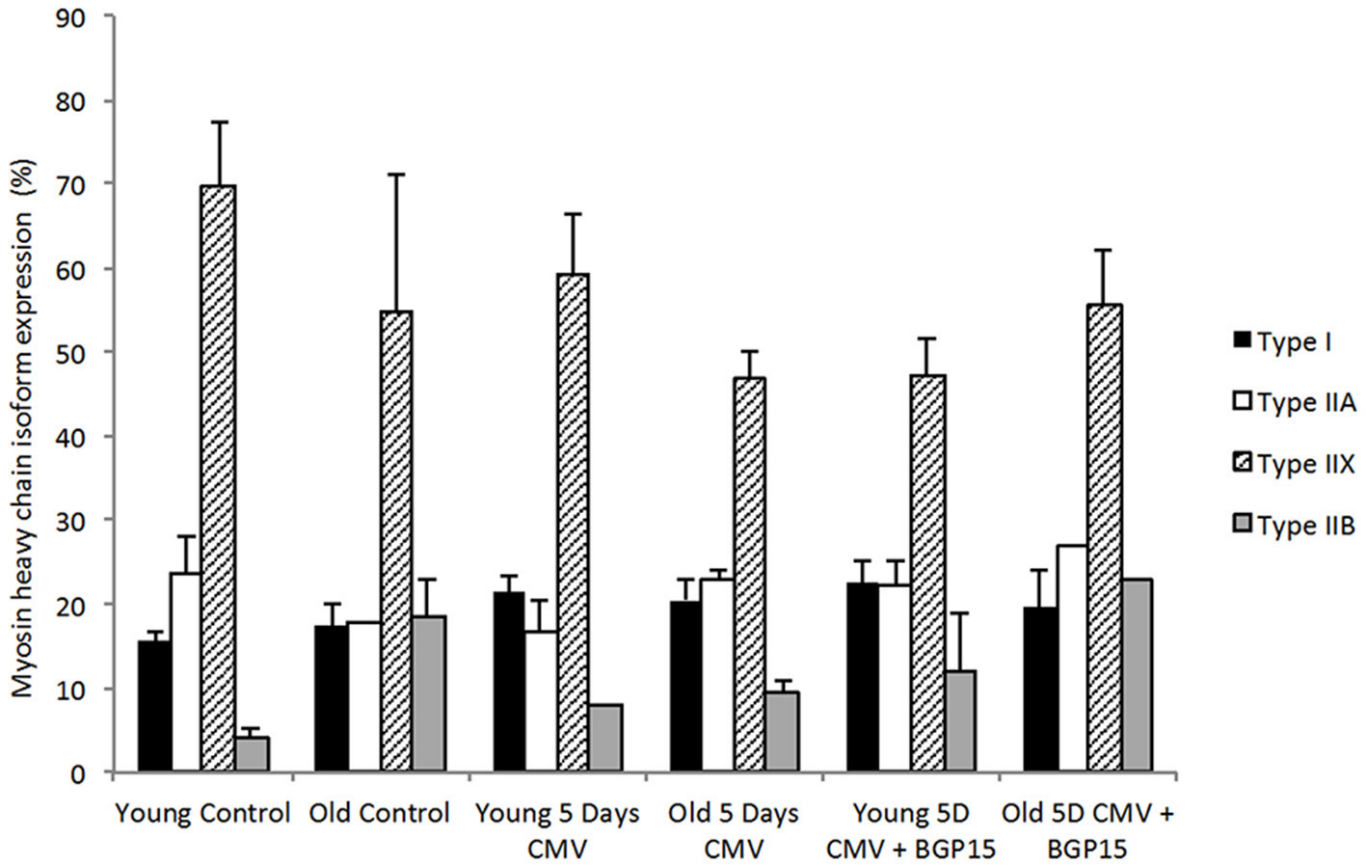
**Myosin heavy chain isoform expression (%) from cross sections in young and old rats in control, 5 days CMV and 5 days CMV+BGP-15 groups**. (Black, type I; white, type IIA; stripe, type IIX; gray, type IIB). Values are % + SEM.

A total of 306 diaphragm muscle fibers met the acceptance criteria and were included in this study. Averages were based on an average of 13 fibers per animal. The fiber cross sectional area (CSA) from the old control animals was significantly larger (*p* < 0.05) than in young adult controls (Figure 2). Five days deep sedation, neuromuscular blockade (NMB) and CMV resulted in a compensatory hypertrophy (*p* < 0.05) in the young. In the old control animals, there is a trend toward an increased CSA, although not statistically significant (Figure 2). BGP-15 administration had no significant effect on muscle fiber size compared with untreated animals during 5 days CMV irrespective animal age (Figure 2). The IIx MyHC isoform was dominant in all groups and there were no statistically significant differences in MyHC isoform expression between groups in response to age, CMV or BGP-15 administration (Figure 3).

### Effects of 5 days CMV with and without BGP-15 on diaphragm single muscle fiber specific and absolute force

**Figure 4:**
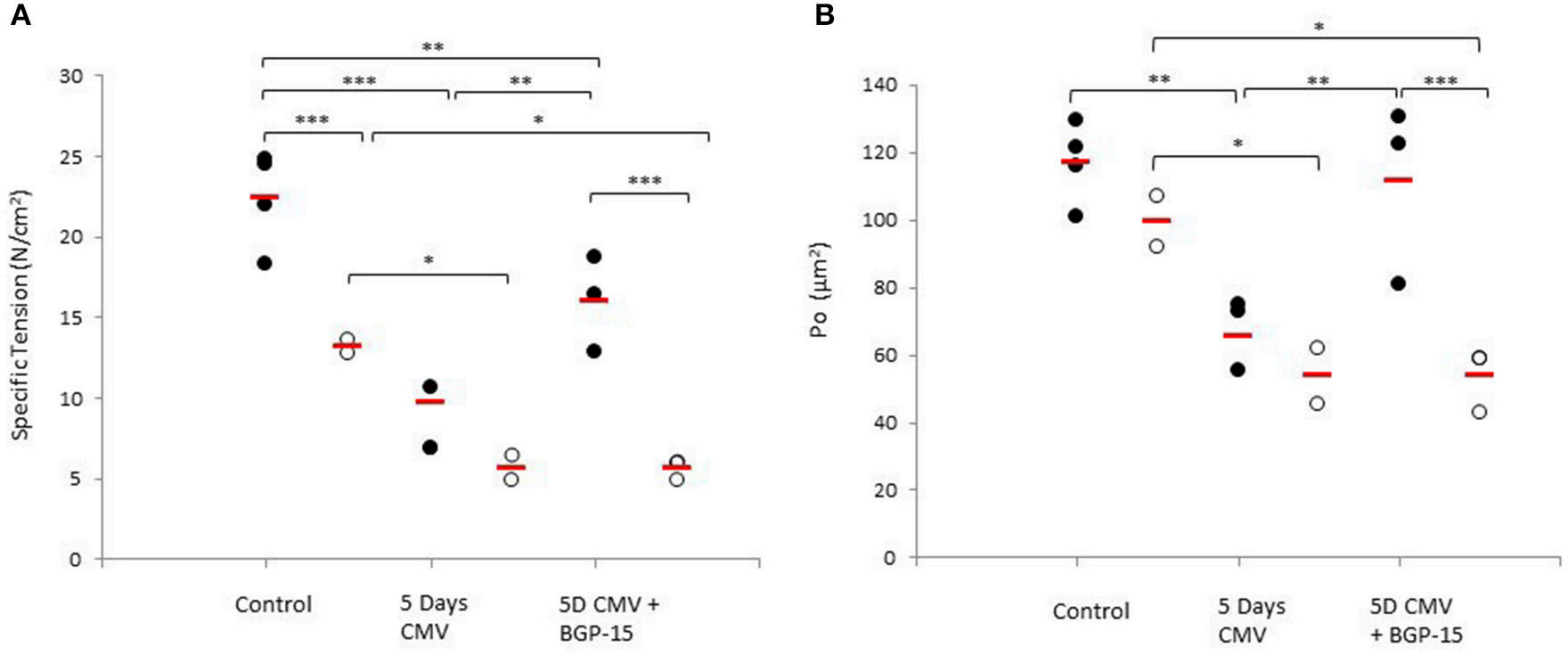
**Individual rat single diaphragm muscle fiber. (A)** specific force (Po/CSA) and **(B)** absolute force (Po) measured at fixed sarcomere length in control animals (individual rats: young *n* = 4 and old *n* = 2) compared with age-matched animals exposed to CMV for 5 days without (individual rats: young *n* = 3 and old *n* = 2) and with BGP-15 (individual rats: young *n* = 3 and old *n* = 3). Values are averages of individual data from the different animals and the red line indicates the average for each group. (Filled circles, young; Open circles, old; red line, group average). Significance level, ^*^p < 0.05, ^**^p < 0.01, ^***^p < 0.001.

An age-related decline in force upon maximum Ca^2+^ activation normalized to muscle fiber CSA (specific force) was observed in controls (*p* < 0.001). In both young and old animals, a significant decrease exceeding 20% (*p* < 0.001 and *p* < 0.01, respectively) was observed in specific force after 5 days mechanical ventilation compared with the control group. Furthermore, systemic administration of BGP-15 had a significant positive effect (*P* < 0.01) on the specific force in the young, but not in the old animals (Figure 4A). Absolute muscle force followed a similar pattern as specific force (Figure 4B).

Cacciani and co-workers (Cacciani et al., 2014) have previously reported that the effects of 5 days CMV on specific force, absolute force and muscle fiber CSA were independent of the MyHC isoform expressed in the fiber. The effects of BGP-15 administration on muscle fiber size, specific and absolute force were also independent of MyHC isoform expression (Table 1).

**Table 1:** **Specific force, cross sectional area (CSA) and absolute force from single diaphragm muscle fibers exposed to 5 days controlled mechanical ventilation + BGP-15, expressing different myosin heavy chain isoforms**.

**Fiber type**	**Specific force (N/cm^2^) ± S.E.M**.		**CSA (μm^2^) ± S.E.M**.		**Po (μN) ± S.E.M**.	
	**Young**	**Old**	**Young**	**Old**	**Young**	**Old**
I (8/13)	18 ± 4	5.2 ± 0.8	882 ± 169	1016 ± 150	128 ± 19	56 ± 6
IIA (2/1)	26 ± 7	8.4	575 ± 125	720	143 ± 10	61
IIB (2/0)	10 ± 2		869 ± 378		82 ± 18	
IIX (7/12)	14 ± 3	5.6 ± 0.9	1023 ± 128	1004 ± 118	124 ± 11	48 ± 4
IIX, I (3/1)	9 ± 1	3.8	606 ± 33	655	59 ± 5.50	25
IIX, IIA (3/2)	20 ± 1	7.7 ± 1.2	531 ± 108	762 ± 20	107 ± 23	59 ± 8
IIX – IIB (1/1)	11 ± 1	7.1	900 ± 194	1032	103 ± 6	74
I-IIA-IIX-IIB (1/1)	11	4.4	1156	1289	132	58

*The values are means ± SEM (young/old), number of fibers per group*.

### Effects of 5 days CMV with and without BGP-15 on Hsp72 expression in diaphragm muscle

**Figure 5:**
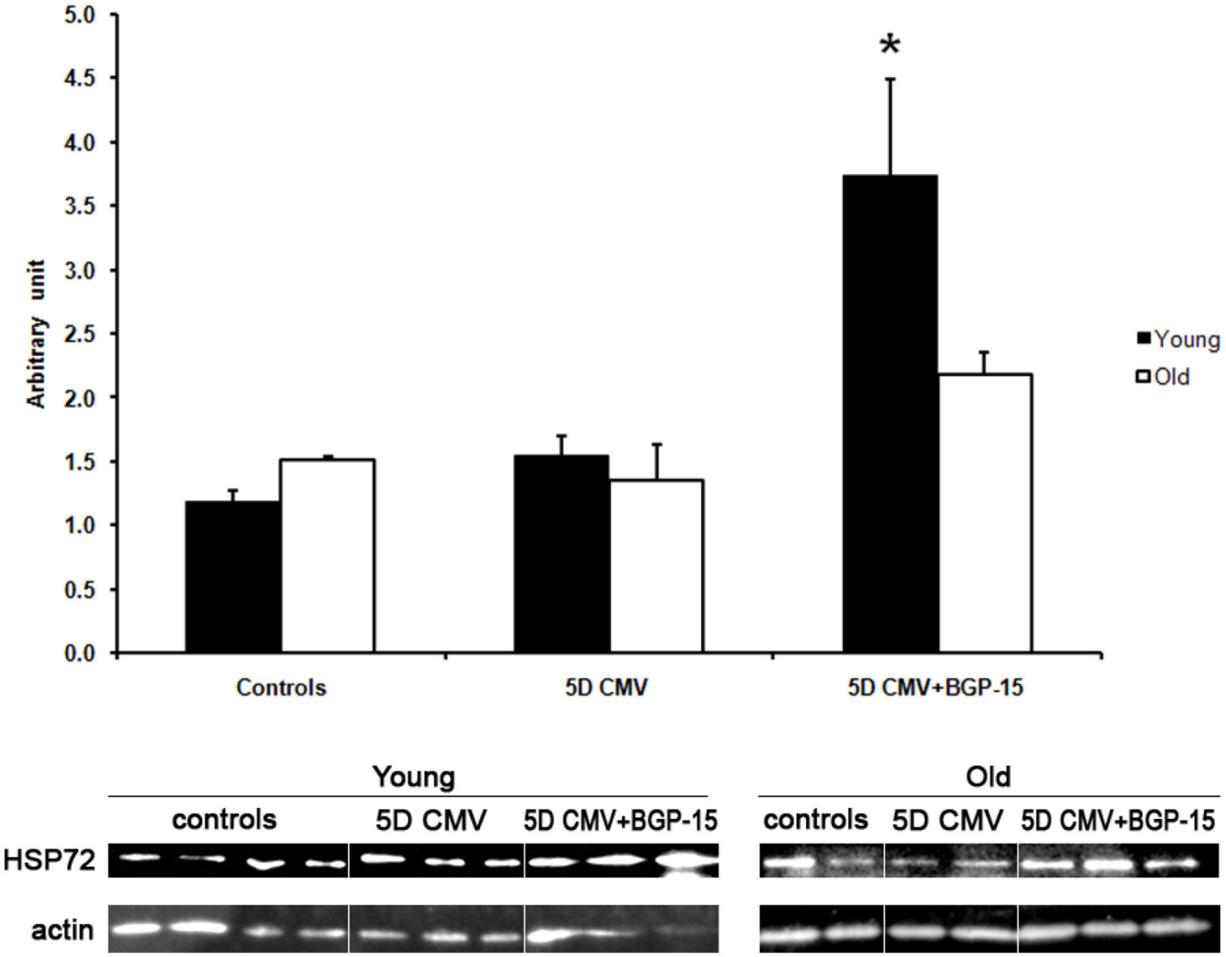
**Western blot analyses of the Hsp72 protein expression normalized to actin contents in the diaphragm in control animals (individual rats: young ***n*** = 4 and old emphn = 2) compared with the age-matched animals exposed to CMV for 5 days with (individual rats: young ***n*** = 3 and old ***n*** = 2) and without BGP-15 (individual rats: young ***n*** = 3 and old ***n*** = 3)**. (Black bars, young; white bars, old). Values are means + SEM. Significance level, ^*^p < 0.05.

Hsp72 protein levels did not differ between young and old animals in the control group or after 5 days CMV. However, the increase (*p* < 0.05) in Hsp72 levels observed in response to 5 days BGP-15 administration in mechanically ventilated rats was restricted to the young animals (Figure 5).

### Effect of 5 days CMV with and without BGP-15 on atrogene mRNA expression

**Figure 6:**
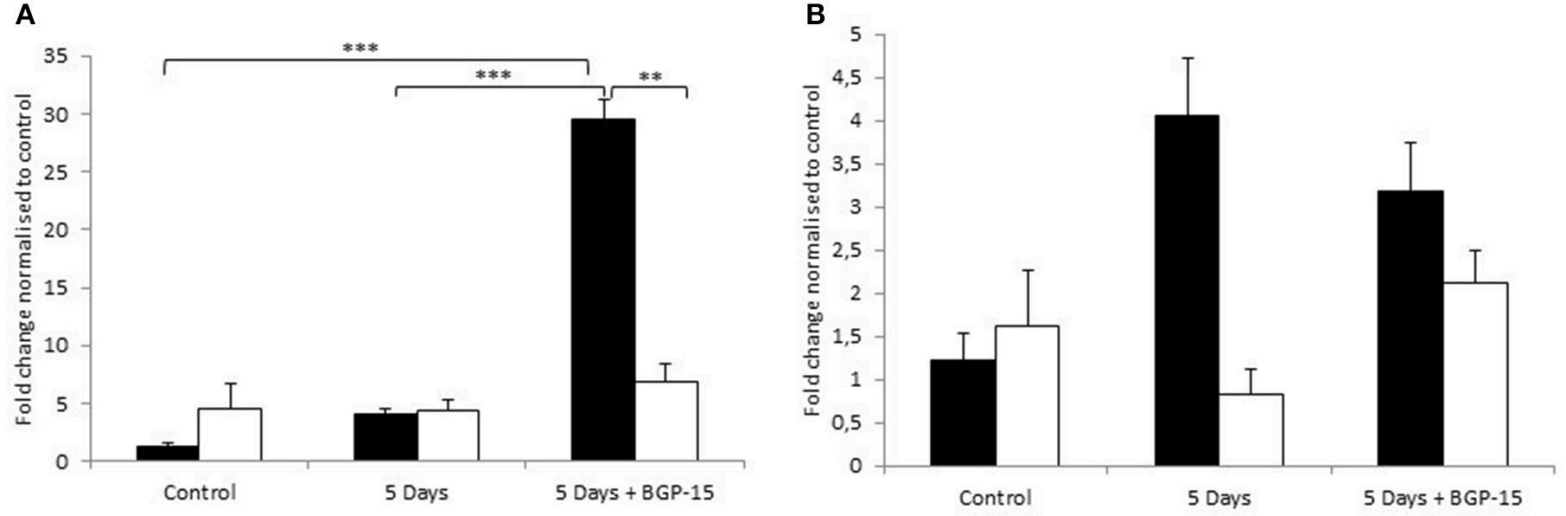
**(A)** MuRF1 gene expression **(B)** Atrogin-1 mRNA expression, according to real time PCR in the Diaphragm. Control animals (individual rats: young *n* = 4 and old *n* = 2) compared with age-matched animals exposed to CMV for 5 days without (individual rats: young *n* = 3 and old *n* = 2) and with BGP-15 (individual rats: young *n* = 3 and old *n* = 3). Values are means of starting quantities + SEM. (Black, young; white, old. Significance level, ^**^p < 0.01, ^***^p < 0.001.

The most striking observation that we made was an increased expression of MuRF1 in only the young in response to 5 days CMV+BGP-15 compared to control values and to 5 days CMV alone (Figure 6A). Furthermore, we show that the transcriptional regulation of MuRF1 and atrogin-1 showed an initial trend of an upregulation after 5 days CMV in the young, however in the old the expression of both atrogene's appeared to be unaltered by 5 days CMV or by BGP-15 (Figures 6A,B). In both instances neither was seen to be statistically different in comparison to control values.

## Discussion

This study is a continuation of previous studies from our group investigating the effects of BGP-15 administration on diaphragm muscle structure and function in young Sprague Dawley rats and aging-related effects of long-term (5 days) CMV on diaphragm muscle function in F344 BN hybrid rats (Cacciani et al., 2014; Salah et al., 2016). The main aim of the present pilot study was to test whether the effects of BGP-15, a Hsp chaperone co-inducer, that alleviates diaphragm contractile dysfunction in young rats, would have a more pronounced beneficial effect in old animals due to a known compromised Hsp protection in old age (Blake et al., 1991; Dodd et al., 2009). In accordance with our hypothesis, BGP-15 had a very strong and positive effect on diaphragm muscle function and Hsp72 expression, but opposite to the original hypothesis this effect was primarily observed in the young and not in the old.

### The effect of CMV and BGP-15 on specific force

A dramatic decline in force production was observed after 5 days of CMV in both the young and old age groups in agreement with previous reports in rats (Powers et al., 1985; Le Bourdelles et al., 1994; Cacciani et al., 2014; Corpeno et al., 2014), baboons (Anzueto et al., 1997) and piglets (Radell et al., 2002; Ochala et al., 2011). This loss of specific force may be attributed to a lower number of myosin cross-bridges per area unit due to the preferential slowing of contractile protein synthesis and/or accelerated destruction of the contractile machinery of the muscle occurring in the ICU (Helliwell et al., 1998; Ochala and Larsson, 2008; Derde et al., 2012; Cohen et al., 2015). It has become more clear that specific ubiquitin ligases play a prominent role (Sandri, 2008) via the muscle ring finger 1 (MuRF1) and muscle atrophy F-box (MAFbx) which greatly promotes the degradation of the molecular motor myosin and related contractile proteins in the ICU and during general disuse conditions (Schiaffino and Mammucari, 2011). Surprisingly we observed an up regulation of MuRF1 gene expression in the young rats after 5 days CMV and BGP-15 treatment in conjunction with an increased force production. To date, MuRF1 and MAFbx mRNA expression have been shown to be elevated at some point during every condition that induces skeletal muscle atrophy (Bodine and Baehr, 2014). In addition, MuRF1 and MAFbx are transiently expressed at the onset of reloading following disuse atrophy (Slimani et al., 2012). Thus, it has become clear that the expression of MuRF1 varies over a time period demonstrating the importance of time-resolved analyses of the atrogenes.

In accordance with our recent findings in young Sprague Dawley rats exposed to 10 days of CMV (Salah et al., 2016), administration of BGP-15 had a significant positive effect on the force-generating capacity of diaphragm muscle fibers irrespective of muscle fiber type. However, this effect was only observed in the young and not in the old animals, i.e., specific force was ~60% lower in the old animals after 5 days of CMV regardless of BGP-15 treatment or not. The restored force generating capacity in the young was associated with an increased Hsp72 expression (Figure 5). BGP-15 may interfere with membrane hyper-structures via their highly specific lipid interactions to enhance stress signals that activate Hsp genes and prolong the binding of HSF-1 to respective DNA elements (Gombos et al., 2011). A vast amount of data support that Hsp's are induced through remodeling cell membranes (Vigh et al., 2007; Gombos et al., 2011). Thus, the up-regulation of Hsp72 may contribute to the maintenance of muscle fiber integrity, facilitating muscle regeneration and recovery (Senf, 2013). In a recently published paper from our group on the pathophysiological mechanisms underlying the impaired specific force in response to CMV in diaphragm muscle fibers from young Sprague Dawley rats, it was clearly demonstrated that the loss of specific force was, at least in part, due to an impaired function of the motor protein myosin due to post-translational protein modifications (PTMs). Furthermore, the loss of force was reversed with BGP-15, in parallel with a partial PTM reversal and myosin function (Salah et al., 2016). Conversely Hsp72 expression is decreased during muscle inactivity and aging and evidence supports the loss of Hsp72 as a key mechanism that may drive muscle atrophy, contractile dysfunction and reduced regenerative capacity associated with these conditions (Singh et al., 2006; Senf et al., 2008). In old rats, BGP-15 failed to activate the potential events described above and it did not promote up-regulation of the induced form of Hsp70, Hsp72 in the diaphragm muscle. However, it cannot be excluded that there is an aging-related shift in the dose-response relationship and an increased amount of BGP-15 may be required to improve diaphragm function old age. Thus, dose-response experiments deserve further scientific attention in the old animals.

### The effect of CMV with the addition of BGP-15 on CSA

In accordance with our previous observation, a compensatory muscle fiber hypertrophy was observed in young and old F344-BN hybrid rats in response to 5 days CMV (Cacciani et al., 2014). Furthermore, we show that BGP-15 had no additive effect to CSA over 5 days CMV in either young or old and that CSA remained constant in this time period. The premise that disuse induces muscle fiber atrophy is widely accepted and has essentially become physiological dogma in the literature. However, this concept has been challenged and there is evidence that the diaphragm does not atrophy as a result of inactivity and an initial, albeit transient, hypertrophy has been reported in diaphragm muscle fibers expressing the type I and IIa MyHC isoforms in response to phrenicotomy or TTX nerve block (Sieck and Mantilla, 2013). The idea of muscle fiber hypertrophy has been alluded to in various models; hypertrophy has been seen previously in a porcine model (Radell et al., 2002), which also demonstrated a decline in *in vivo* diaphragm function. Kavazis *et al* (Kavazis et al., 2007) also showed that in male F344-BN rats aging resulted in an increase in the CSA of type IIA fibers. Furthermore, it has been suggested that both hypertrophy and atrophy may occur in skeletal muscle with the aging process. Moreover, a study undertaken in cardiac tissue has demonstrated a similar pattern (atrophy and hypertrophy) in senescent hearts of F344-BN rats (Wanagat et al., 2002). This can be matched to our similar finding in the diaphragm, being consistent with the idea that this pattern occurs in muscles that remain highly activated with aging. Pardo and co-workers suggested that the age related increases in muscle stiffness might be responsible for the altered response of the AKT and IKK signaling pathways to the mechanical stimulation, which can in turn affect the FoXO1 and FoXO activity (Pardo et al., 2008). Thus, these changes may be related to age and CMV changes in mechano-sensing and mechano-transduction pathways.

It is established that Hsp70 has an intracellular effect on the inhibition of nuclear factor κB (NF-κB) activation, which has profound implications for immunity, inflammation, cell survival and apoptosis. Hsp70 blocks NF-κB activation at different levels. The inhibition of these transcriptional pathways may explain the prevention of muscle-fiber atrophy by Hsp70, since NF-κB and FoxO are independently sufficient to cause skeletal muscle atrophy (Sandri et al., 2004). Furthermore, the inhibitory potential of Hsp70 over apoptosis occurs via many different intracellular downstream pathways (e.g., JNK, NF-κB and Akt), which are directly and indirectly blocked by Hsp70. Collectively, these mechanisms underlie Hsp70 anti-apoptotic effects in cells under stress conditions (Thiago Gomes Heck et al., 2012). This and further evidences suggest that Hsp70 inhibits key signaling pathways for muscle atrophy (Thiago Gomes Heck et al., 2012) and potentially be an explanation for the hypertrophy demonstrated. The hypertrophy is not incompatible with variations in the contractile protein contents (described in the previous section) but probably means a change in the inter-myofilament spacing or a change in the amount of myoplasma with preserved inter-myofilament spacing.

### Study limitation and future work

The small number of animals in this study limits the detection of significant changes in the population, i.e., type II error. The labor intense and technically difficult experimental rat model mimicking the ICU condition, requiring extensive monitoring 24 h per day during the 5-day experimental period, and the difficulty in obtaining old F344-BN hybrid animals has limited the number of animals included in the young and old groups. This limitation is acknowledged and a confirmatory follow up study is needed in a larger population of animals. However, we put forward from the present results that we have ascertained a valid model for further investigating this intervention and to applying it to a highly relevant and necessary topic of research.

In addition to increasing the total number of rats, future work would have to focus on the molecular mechanisms underlying the differences we found in the present study. First we will have to clearly understand why specific force is restored in BGP-15 treated young rats and why this does not occur in old rat. As is mentioned previously in a recently published paper from our group the role of PTM has become evident as to playing a major role and thus it would be advantageous to further investigate the role in relation to the F344-BN hybrid rats and the impact of aging.

## Conclusion

Significant age-specific differences were observed in control diaphragm fibers and in the response to 5 days CMV. Further, BGP-15 had a strong positive effect on diaphragm muscle fiber function and Hsp72 expression after 5 days CMV, but this effect was restricted to the young animals. The underlying mechanisms remain to be studied.

## Author contributions

The experimental work was performed in the research laboratory of LL. The study was designed and supervised by LL. Protein expression was carried out by HA. HO carried out qRT-PCR. NC and HO carried out contractile measurements of single muscle fibers and wrote the manuscript with assistance from LL. All authors read and approved the final manuscript.

## Funding

The study was supported by grants from the Swedish Research Council (8651), the Swedish Foundation for International Cooperation in Research and Higher Education (STINT), King Gustaf V and Queen Victoria's Foundation and the European Commission (MyoAge, EC Fp7 CT-223756 and COST CM1001) to LL.

### Conflict of interest statement

The authors declare that the research was conducted in the absence of any commercial or financial relationships that could be construed as a potential conflict of interest.
